# Effects of Voice Therapy on Vocal Tract Discomfort in Muscle Tension Dysphonia

**Published:** 2019-09

**Authors:** Banafshe Mansuri, Farhad Torabinezhad, Ali-Ashraf Jamshidi, Payman Dabirmoghadam, Behnoosh Vasaghi-Gharamaleki, Leila Ghelichi

**Affiliations:** 1 *Department of Speech and Language Pathology, School of Rehabilitation Sciences, Iran University of Medical Sciences, Tehran, Iran.*; 2 *Department of Physical Therapy, School of Rehabilitation Sciences, Iran University of Medical Sciences, Tehran, Iran.*; 3 *Otolaryngology Research Center, Tehran University of Medical Science, Tehran, Iran.*; 4 *Department of Basic Sciences in Rehabilitation, School of Rehabilitation Sciences, Iran University of Medical Sciences, Tehran, Iran.*

**Keywords:** Pain, Therapy, Voice, Voice disorders, Voice quality

## Abstract

**Introduction:**

Patients with muscle tension dysphonia (MTD) suffer from several physical discomforts in their vocal tract. However, few studies have examined the effects of voice therapy (VT) on the vocal tract discomfort (VTD) in patients with voice disorders. Therefore, the aim of the present study was to investigate the effects of VT on the VTD in patients with MTD.

**Materials and Methods:**

This study was carried out on 25 subjects with MTD, including 5 men and 20 women, with the mean age of 37.20±5.70 years. The participants underwent 10 consecutive sessions of VT twice a week. The acoustic voice analysis, auditory-perceptual assessment, and the Persian version of the vocal tract discomfort (VTDp) scale were used to compare the pre- and post-treatment results.

**Results:**

After VT, significant improvements were observed in the acoustic characteristics, including jitter, shimmer, and harmonics-to-noise ratio (P<0.05). Regarding the auditory-perceptual assessment, a significant reduction was noticed in the overall severity, roughness, and breathiness (P<0.05). Moreover, VT led to a significant reduction in all the items of the VTDp, including burn, tightness, dryness, pain, tickling, soreness, irritability, and lump in the throat, after VT in both frequency and severity sections of the VTDp scale (P<0.05).

**Conclusion:**

The results of the present study showed that VT can be effective in reducing the frequency and severity of the VTD in patients with MTD in addition to improving voice quality.

## Introduction

Muscle tension dysphonia (MTD) is one of the most common voice disorders that is very prevalent in laryngology clinics (1). According to the statistics,10-40% of patients referred to voice clinics are MTD cases (2,3). In this pathological condition, the tension increased in both groups of intrinsic and extrinsic muscles of the larynx (2,4-7). 

The vocal abuse and misuse, as well as psychological/personality factors, can be among the main creators of MTD. Sometimes the presence of an underlying disease, such as lesions in vocal cords, infection in the upper respiratory tract, and laryngopharyngeal reflux, can lead to MTD due to the patient's attempt to compensate for these underlying diseases (2,6). 

Primary and secondary MTDs are two types of MTD. The presence or absence of an organic lesion determines the type of MTD. When there is no organic lesion, it is called primary MTD; otherwise, the MTD is a secondary type (2,6,8). In order to confirm the presence of MTD, some important points should be considered during the evaluation of the patient’s history, including voice abuse/misuse, psychological factors, and stressful conditions. The presence of tightness and significant tension in the (para) laryngeal muscles decreased space of the thyrohyoid and cricothyroid membranes, laryngeal rise, and local tenderness during phonation. Subsequently, the visual assessment of the larynx should be performed using videostroboscopy (6,9).

Patients with MTD suffer from vocal fatigue, voice loss, hoarseness, vocal strain, pain, and neck tightness (1,4,8,10). Moreover, based on the literature and clinical experiences, most MTD patients reported the vocal tract discomfort (VTD) as one of their symptoms (6,11-13). These VTD symptoms included the sensation of the items in the vocal tract as follows: burn, tightness, dryness, pain, tickling, soreness, irritability, and lump in the throat. Due to the high prevalence of the VTD symptoms in MTD, as well as its high negative impacts on the patients’ life, the VTD scale was developed to assess discomfort symptoms in the vocal tract in patients with voice disorders (14). However, the VTD is reported in most MTD patients, and the existence of the VTD scale as a valid reliable and easy-to-use scale to evaluate the VTD, few studies applied this scale as an outcome measurement for investigating the effectiveness of voice therapy (VT) in MTD patients. Most of the studies that investigated the effectiveness of VT for MTD used outcome measurements, such as acoustic voice analysis, auditory perceptual evaluation, and laryngeal examination. 

Moreover, a limited number of studies has investigated the effectiveness of VT in the VTD symptoms. For example, Jafari et al. reported the effect of vocal function exercises on MTD (10). They used the auditory-perceptual evaluation and the Voice Handicap Index (VHI) as outcome measurements. It was observed that vocal function exercises can cause significant improvements in the VHI and auditory-perceptual evaluation (10). 

Watts et al. investigated the effectiveness of Stretch-and-Flow (SnF) VT in patients with MTD. The VHI questionnaire, as well as aerodynamic and acoustic parameters, were applied to evaluate the treatment effects. It was observed that SnF method in comparison with vocal hygiene resulted in a better significant decrease in the VHI, as well as significant improvements in the maximum phonation time and acoustic analysis (15). 

Another study conducted by Sielska-Badurek et al. regarding the effects of functional VT for singers with MTD assessed the treatment effects with VHI, laryngostroboscopy, palpation, as well as perceptual and acoustic assessments. After 10-15 sessions of treatment, they observed a great improvement in the vocal palpation, as well as perceptual and acoustic assessments (1). Dehqan and Scherer investigated the long-term effects of manual circumlaryngeal therapy on the treatment of MTD. They observed the improvement of acoustic parameters, including jitter, shimmer, and harmonic to noise ratio. 

Moreover, in the aforementioned study, they observed that the treatment caused positive effects on auditory-perceptual evaluation (16). To the best of our knowledge, only a unique study carried out by Mathieson et al. used the VTD assessment to investigate the effects of VT on MTD patients. In the aforementioned study, it was observed that laryngeal manual therapy is effective in reducing the frequency and severity of the VTD (6). Overall, most studies that investigated the effects of VT on MTD have used outcome measurements other than the VTD. Therefore, the aim of the present study was to investigate the effects of VT on the VTD in patients with MTD.

## Materials and Methods

**Participants**

A total of 25 patients with primary MTD (20 women and 5 men) participated in the present study. The mean age of the participants was reported as 37.20±5.70 years. The patients were recruited from the Ear, Nose, and Throat Department of Amir A’lam Hospital of Tehran, Iran. To diagnose MTD, the case history-taking, musculoskeletal evaluation, and laryngeal evaluation with videostroboscopy were conducted by an otolaryngologist and an experienced speech-language pathologist (SLP). 

The inclusion criteria were the patients with primary MTD, within the age range of 18-45 years with normal speech and language, as well as no history of neurological problems, hearing defects, previous laryngeal surgery, hormone or thyroid deviation, and vascular or cardiologic disorders. Furthermore, the exclusion criteria were pregnancy, smoking, alcohol consumption, and acute or chronic upper respiratory infection during the treatment. To assess the inclusion and exclusion criteria, each participant was examined by an otolaryngologist. The patients who met the inclusion criteria received 10 consecutive sessions of VT twice a week (each session: 45 min). The time of each intervention session was based on a study conducted by Stemple (1993) and previous studies (1,10,14). The acoustic voice analysis, auditory-perceptual assessment, and Persian version of the vocal tract discomfort (VTDp) scale were used to compare the results before and after the treatment.

**Evaluation**

**Acoustic voice analysis**

The vowel /a/ was used in acoustic voice analysis. The participants were instructed to sustain three stable /a/ vowels for a minimum of 5 sec at their habitual pitch and loudness, with about 5 sec interval between each repetition. The voice samples were collected in a sound-treated room. A Zoom H5 handy digital recorder (Zoom Corporation, Tokyo, Japan) was used with a microphone capsule. The digital recorder H5 provides two unidirectional condenser microphones set at a 90-degree angle placed on a stand at a distance of 30 cm in front of the patient’s mouth. 

The voice samples were recorded with a 44.1-kHz sampling frequency and 16-bit resolution. The final (third) repetition of the vowel samples was used for the analysis (17); the middle three seconds of voice samples was used for acoustic analysis. The F0 (Hz), jitter (%), shimmer (%), and harmonics-to-noise ratio (HNR) (dB) were the acoustic parameters extracted using Praat software (version 6.0.23; University of Amsterdam., Amsterdam, Netherlands) (18).

**Auditory-perceptual assessment**

The Persian version of the Consensus Auditory-perceptual Evaluation of Voice (CAPE-V) scale was used for auditory-perceptual assessment (19). The CAPE-V is a subjective scale to evaluate voice quality based on several tasks. The CAPE-V included voice breathiness, roughness, loudness, pitch, strain, and overall severity parameters. The score of each parameter is based on a 100 mm visual analog scale, with 0 and 100 scores indicating normal voice and severely impaired voice, respectively (12,19). 

The parameters of CAPE-V used in the present study included overall severity, roughness, and breathiness. The sustained vowels, sentence reading, and patients' connected speech were rated by two SLPs with at least 5 years of experience in the field of VT. The voice samples (before and after the treatment) of each patient were randomly given to the raters in a quiet room for evaluation using the CAPE-V. It should be noted that the raters were blind to the objectives and procedure of the study.

**Vocal Tract Discomfort**

The VTD of each participant was assessed using the VTDp scale. The VTDp scale validated by Torabi et al. (20) was proven to have normal validity and reliability. The VTDp scale consists of two sections that quantify the frequency and severity of physical discomfort in the vocal tract; each section includes the items as follows: burn, tightness, dryness, pain, tickling, soreness, irritability, and lump in the throat. The frequency and severity of the items were separately rated by the patients using a seven-point Likert scale from zero to six. Given that each section includes eight items, and each item’s score is from zero to six, the overall score for each section (frequency of severity) is at least 0 and up to 48 (6,20). In the present study, each participant was instructed to complete the VTDp scale before and after the treatment.

**Voice Therapy**

According to the evidence regarding the treatment of MTD, both indirect and direct VTs were used in the present study for the treatment of MTD. The indirect VT included the patient’s education and vocal hygiene (2). The vocal hygiene program used in the present study was similar to a vocal hygiene program used by Chan and Rodríguez-Parra et al. in previous studies (21,22). This vocal hygiene program included descriptions about normal voice mechanisms and voice disorders, vocal abuse/misuse and its consequences, correct and proper (healthy) use of voice, and some personalized strategies (21,22). The VT techniques used in direct VT included abdominal breathing training, chewing, yawn-sigh, laryngeal manual therapy, and tongue trills (6,23-26). In each treatment session, VT techniques were used in combination with each other’s or independently due to the patient’s needs (27).

**Ethical consideration**

The present study was approved by the Ethics Committee affiliated to Iran University of Medical Sciences (IR.IUMS. FMD. REC 1396. 9321363002). Participation in the study was voluntary, and the participants could withdraw at any stage of the study. All the subjects completed an informed consent form. Moreover, there was no charge for the treatment of the participants.

**Statistical analysis**

In this study, the Kolmogorov-Smirnov test was used to investigate the normality of the data. The paired sample t-test was used to compare the variables before and after the treatment. SPSS software (version 20.0) was utilized to perform the statistical analysis. The significance level was set at P<0.05 for all the statistical tests

## Results

**Acoustic voice analysis**

The comparison of the acoustic parameters showed that there were significant improvements in jitter, shimmer, and HNR before and after VT. Table 1 tabulates the results of acoustic parameters before and after the treatment.

**Table 1:** Comparison of acoustics parameters before and after voice therapy; n=25

Sustained /a/	Before voice therapy	After voice therapy	Difference	P-value
**F0 (Hz)**	202.87 (44.02)	203.50 (43.95)	+0.721	0.821
**Jitter (%)**	0.761 (0.76)	0.401 (0.18)	-0.359	0.03
**Shimmer (%)**	5.04 (3.34)	2.94 (1.15)	-2.09	<0.006
**Harmonics-to-noise ratio** ** (dB)**	19.05 (4.52)	21.6 (3.57)	+2.54	0.013

Measurements of paired sample t-test and mean±standard deviation before and after treatment

*Auditory-perceptual assessment*

The comparison of auditory-perceptual assessment results using the CAPE-V showed that there were significant improvements in overall severity, roughness, and breathiness parameters of the CAPE-V before and after VT. 

Table 2 shows the results of the CAPE-V before and after the treatment.

**Frequency of Vocal Tract Discomfort**

***The comparison of the frequency section of the VTDp scale before and after VT showed that there was a statistically significant reduction in some items as follows: burn, tightness, dryness, pain, tickling, soreness, irritability, and lump in the throat. The total score of the frequency of the VTDp decreased from 26.12 to 8.76 after VT. More details about the frequency section of the VTDp scale are presented in*** Table 3 ***.***

**Table 2:** Comparison of auditory-perceptual assessment (CAPE-V) before and after voice therapy; n=25

CAPE-V parameters	Before voice therapy	After voice therapy	Difference	P-value
**Overall severity**	53 (12.29)	34.5 (10.74)	-18.5	<0.000
**Roughness**	49.5 (12.76)	32.25 (10.06)	-17.25	<0.000
**Breathiness**	49 (13.03)	31.5 (11.25)	-17.5	<0.000

Measurements of paired sample t-test and mean±standard deviation before and after treatment

CAPE-V: Consensus auditory-perceptual evaluation of voice

**Table 3:** Comparison of frequency of VTDp before and after voice therapy; n=25

Items	Before voice therapy	After voice therapy	Difference	P-value
Burning	2.80 (2.14)	0.92 (1.28)	-1.88	P<0.000
Tightness	3.28 (2.15)	1.28 (1.54)	-2.00	P<0.000
Dryness	4.68 (1.60)	1.32 (1.40)	-3.36	P<0.000
Pain	3.72 (2.09)	0.96 (1.02)	-2.76	P<0.000
Tickling	1.44 (1.89)	0.44 (0.82)	-1.00	P<0.010
Soreness	2.92 (2.23)	1.04 (1.36)	-1.88	P<0.000
Irritability	4.00 (1.87)	1.72 (1.54)	-2.28	P<0.000
Lump in throat	3.40 (2.25)	1.08 (1.07)	-2.32	P<0.000
Total Score of VTD	26.12 (7.21)	8.76 (6.32)	-17.36	P<0.000

Measurements of paired sample t-test and mean±standard deviation before and after treatment

VTDp: Persian version of vocal tract discomfort, VTD: Vocal tract discomfort

**Severity of Vocal Tract Discomfort**

The comparison of the severity section of the VTDp scale before and after VT showed that there was a statistically significant reduction in the items as follows: burn, tightness, dryness, pain, tickling, soreness, irritability, and lump in the throat. Moreover, the total score of the severity of the VTDp decreased from 27.88 to 9.28 after VT. More details about the severity section of the VTDp scale are presented in (Table.4).

**Table 4:** Comparison of severity of VTDp before and after voice therapy; n=25

Items	Before voice therapy	After voice therapy	Difference	P-value
**Burn**	3.32 (2.30)	1.04 (1.45)	-2.28	P<0.000
**Tightness**	3.64 (2.32)	1.40 (1.65)	-2.24	P<0.000
**Dryness**	4.84 (1.51)	1.32 (1.43)	-3.52	P<0.000
**Pain**	3.72 (2.05)	1.04 (1.20)	-2.68	P<0.000
**Tickling**	1.68 (2.17)	0.52 (1.04)	-1.16	P<0.007
**Soreness**	3.00 (2.32)	1.00 (1.38)	-2.00	P<0.000
**Irritability**	3.92 (1.91)	1.60 (1.55)	-2.32	P<0.000
**Lump in throat**	3.72 (2.39)	1.08 (1.07)	-2.64	P<0.000
**Total Score of VTD**	27.88 (7.35)	9.28 (7.05)	-18.60	P<0.000

Measurements of paired sample t-test and mean±standard deviation before and after treatment

VTDp: Persian version of vocal tract discomfort,     VTD: Vocal tract discomfort

## Discussion

Patients with MTD have some problems, including vocal fatigue, voice loss, hoarseness, vocal strain, pain, neck tightness, and VTD. Given that the VTD is common among MTD patients and have negative effects on patients’ life, using the VTD assessment to evaluate the effectiveness of treatment can be important. However, few studies have used the VTD as an outcome measurement to examine the effects of treatment on MTD. Therefore, the aim of the present study was to investigate the effects of VT on the VTD in patients with MTD.

In the present study, VT caused significant improvements in the acoustic parameters, including jitter, shimmer, and HNR. The results of previous studies that used VT for the treatment of MTD have shown that VT could cause significant improvements in the acoustic parameters (8,11,15,28, 29). For example, Roy et al. reported the positive effects of manual circumlaryngeal therapy on jitter and shimmer (11). In a study carried out by de Oliveira Lemos et al., the acoustic parameters, including jitter and shimmer, reduced after manual therapy in MTD patients (8). 

The above-mentioned results showed that VT can improve the vocal fold vibratory behavior that leads to positive changes on the acoustic parameters (8). Furthermore, the results of the present study showed significant positive changes on auditory-perceptual evaluations following VT. The findings of the present study regarding the perceptual assessment are in line with the results of previous studies (8, 10,29,30). These changes in perceptual assessment can be related to improved voice projection and sound emission stability achieved by VT (8,23).

Regarding the VTD, VT caused a significant reduction in all the items of the VTDp scale (both frequency and severity section). These results showed that VT is effective in decreasing the physical discomfort in the vocal tract reported by patients with MTD. The presence of the VTD in MTD cases may be related to an increased tension that exists in the laryngeal musculature (both intrinsic and extrinsic muscles) (20). Therefore, decreasing the laryngeal tension is necessary to reduce the VTD in MTD patients. Both indirect and direct VTs were used in the present study, and each of these two methods can reduce muscle tension. The indirect VT can remove vocal abuse or misuse that may be an underlying cause of muscle tension. Moreover, VT techniques, such as laryngeal manual therapy, yawn-sigh, and other techniques applied in the present study, can decrease tension in intrinsic and extrinsic laryngeal muscles. 

For example, laryngeal manual therapy can directly reduce the tension of the extrinsic laryngeal muscle (6), while another technique, such as yawn-sigh, can affect the intrinsic laryngeal muscles (23). Therefore, the VTD reduction in the present study may be explained by decreasing the laryngeal tension achieved by VT. In this regard, the findings of the present study are in line with the results of a study carried out by Mathieson et al. (6). They reported that laryngeal manual therapy is effective in reducing the frequency (dryness, tickling, soreness, and irritability) and severity (tightness, dryness, pain, and soreness) of VTD. This reduction in VTD items was obtained in a single-session use of laryngeal manual therapy (6). 

The obtained results of the present study were better than those of a study conducted by Mathieson et al. (6). This could be due to more treatment sessions and more VT techniques that were used in the present study. In another study carried out by Woźnicka et al., the applicability of the VTD was evaluated with investigating the effects of VT on teachers with dysphonia. It was observed that all the items of the frequency and severity of VTD significantly reduced after the treatment. 

The VT program used in the aforementioned study, similar to the present study, included both direct and indirect VTs. Moreover, their VT plan was longer than one session and lasted 3 to 4 months (13). It can be concluded that the VTD can decrease in MTD patients even with a single session of VT, which included laryngeal manual therapy; however, better results can be obtained with more sessions of VT. Further studies can provide more accurate statements in this regard.

It should be noted that a complete assessment of patients with voice disorders should include auditory-perceptual assessment, acoustic voice analysis, visual laryngeal evaluation, and self-evaluation (31,32). In some voice disorders, such as MTD, self-evaluation is very important. In these subjects, there is not necessarily a clear relationship between self-evaluations, such as VTD and other assessments (2,33). For example, sometimes a small change in the voice of these patients leads to a lot of changes in self-evaluation scores or vice versa (33). Therefore, the consideration of this evaluation component as an outcome measurement, such as VTD, in MTD patients is very important.

In the present study, long-term assessments were not used, such as a 1-month or 3-month follow-up. In order to better understand the effects of VT on VTD in patients with MTD, future studies should consider this issue. Given that the results of the present study demonstrated that VT caused improvements in VTD symptoms in MTD patients, future studies are suggested to examine the effects of various VT programs on VTD in patients with MTD.

## Conclusion

The obtained results of the present study showed that VT can cause positive significant changes in VTD symptoms in cases with MTD in addition to positive changes that VT makes in acoustic and perceptual evaluations. Therefore, using VT with both direct and indirect VT techniques is a good option for the treatment of VTD in MTD patients. Given that the present study focused on the VTD symptoms, further studies with large sample size and more evaluations are required to make better comments about the effectiveness of VT in MTD.

## References

[1] Sielska-Badurek E, Osuch-Wójcikiewicz E, Sobol M, Kazanecka E, Rzepakowska A, Niemczyk K (2017). Combined functional voice therapy in singers with muscle tension dysphonia in singing. Journal of Voice.

[2] Van Houtte E, Van Lierde K, Claeys S (2011). Pathophysiology and treatment of muscle tension dysphonia: a review of the current knowledge. Journal of Voice.

[3] Van Houtte E, Van Lierde K, D'haeseleer E, Claeys S (2010). The prevalence of laryngeal pathology in a treatment‐seeking population with dysphonia. The Laryngoscope.

[4] Altman KW, Atkinson C, Lazarus C (2005). Current and emerging concepts in muscle tension dysphonia: a 30-month review. Journal of Voice.

[5] Kunduk M, Fink DS, McWhorter AJ (2016). Primary Muscle Tension Dysphonia. Current Otorhinolaryngology Reports.

[6] Mathieson L, Hirani S, Epstein R, Baken R, Wood G, Rubin J (2009). Laryngeal manual therapy: a preliminary study to examine its treatment effects in the management of muscle tension dysphonia. Journal of Voice.

[7] Casper JK, Leonard R (2006). Understanding voice problems: A physiological perspective for diagnosis and treatment.

[8] de Oliveira Lemos I, da Cunha Pereira G, SantAnna GD, Cassol M (2017). Effects of a Voice Therapy Program for Patients with Muscle Tension Dysphonia. Folia Phoniatrica et Logopaedica.

[9] Mansuri B, Torabinejhad F, Jamshidi AA, Dabirmoghaddam P, Vasaghi-Gharamaleki B, Ghelichi L (2018). Transcutaneous Electrical Nerve Stimulation Combined With Voice Therapy in Women With Muscle Tension Dysphonia. Journal of Voice.

[10] Jafari N, Salehi A, Izadi F, Moghadam ST, Ebadi A, Dabirmoghadam P (2016). Vocal Function Exercises for Muscle Tension Dysphonia: Auditory-Perceptual Evaluation and Self-Assessment Rating. Journal of Voice.

[11] Roy N, Bless DM, Heisey D, Ford CN (1997). Manual circumlaryngeal therapy for functionaldysphonia: An evaluation of short-and long-term treatment outcomes. Journal of Voice.

[12] Kempster GB, Gerratt BR, Abbott KV, Barkmeier-Kraemer J, Hillman RE (2009). Consensus auditory-perceptual evaluation of voice: development of a standardized clinical protocol. American Journal of Speech-Language Pathology.

[13] Woźnicka E, Niebudek-Bogusz E, Kwiecień J, Wiktorowicz J, Śliwińska-Kowalska M (2012). Applicability of the vocal tract discomfort (VTD) scale in evaluating the effects of voice therapy of occupational voice disorders. Med Pr.

[14] Stemple JC (1993). Voice therapy: clinical case studies: St.

[15] Watts CR, Hamilton A, Toles L, Childs L, Mau T (2015). A randomized controlled trial of stretch-and-flow voice therapy for muscle tension Dysphonia. The Laryngoscope.

[16] Sluka K, Vance C, Lisi T (2005). High‐frequency, but not low‐frequency, transcutaneous electrical nerve stimulation reduces aspartate and glutamate release in the spinal cord dorsal horn. Journal of neurochemistry.

[17] Dejonckere PH, Bradley P, Clemente P, Cornut G, Crevier-Buchman L, Friedrich G (2001). A basic protocol for functional assessment of voice pathology, especially for investigating the efficacy of (phonosurgical) treatments and evaluating new assessment techniques. European Archives of Oto-rhino-laryngology.

[18] Paul B, Weenink D doing phonetics by computer (Version 6.0.23) [Computer program].

[19] Salary Majd N, Maryam Khoddami S, Drinnan M, Kamali M, Amiri-Shavaki Y, Fallahian N (2014). Validity and rater reliability of Persian version of the Consensus Auditory Perceptual Evaluation of Voice. Audiology.

[20] Torabi H, Khoddami SM, Ansari NN, Dabirmoghaddam P The Vocal Tract Discomfort Scale: validity and reliability of the Persian version in the assessment of patients with muscle tension dysphonia. Journal of Voice.

[21] Rodríguez-Parra MJ, Adrián JA, Casado JC (2011). Comparing voice-therapy and vocal-hygiene treatments in dysphonia using a limited multidimensional evaluation protocol. Journal of communication disorders.

[22] Chan RWK (1994). Does the voice improve with vocal hygiene education? A study of some instrumental voice measures in a group of kindergarten teachers. Journal of Voice.

[23] Boone DR, McFarlane SC, Von Berg SL, Zraick RI (2005). The voice and voice therapy.

[24] Meerschman I, D’haeseleer E, Catry T, Ruigrok B, Claeys S, Van Lierde K (2017). Effect of two isolated vocal facilitating techniques glottal fry and yawn-sigh on the phonation of female speech-language pathology students: A pilot study. Journal of communication disorders.

[25] Van Lierde KM, De Ley S, Clement G, De Bodt M, Van Cauwenberge P (2004). Outcome of laryngeal manual therapy in four Dutch adults with persistent moderate-to-severe vocal hyperfunction: a pilot study. Journal of Voice.

[26] Khatoonabadi AR, Khoramshahi H, Khoddami SM, Dabirmoghaddam P, Ansari NN (2018). Patient-Based Assessment of Effectiveness of Voice Therapy in Vocal Mass Lesions with Secondary Muscle Tension Dysphonia. Iranian journal of otorhinolaryngology.

[27] Craig J, Tomlinson C, Stevens K, Kotagal K, Fornadley J, Jacobson B (2015). Combining voice therapy and physical therapy: a novel approach to treating muscle tension dysphonia. Journal of communication disorders.

[28] Van Lierde KM, De Bodt M, Dhaeseleer E, Wuyts F, Claeys S (2010). The treatment of muscle tension dysphonia: a comparison of two treatment techniques by means of an objective multiparameter approach. Journal of Voice.

[29] Dehqan A, Scherer RC (2018). Positive Effects of Manual Circumlaryngeal Therapy in the Treatment of Muscle Tension Dysphonia (MTD): Long Term Treatment Outcomes. Journal of Voice.

[30] Van Lierde K, Claeys S, De Bodt M, Van Cauwenberge P (2007). Long-term outcome of hyperfunctional voice disorders based on a multiparameter approach. Journal of Voice.

[31] Stemple JC, Roy N, Klaben BK (2014). Clinical voice pathology: Theory and management: Plural Publishing.

[32] Mansuri B, Tohidast SA, Soltaninejad N, Kamali M, Ghelichi L, Azimi H (2018 ). Nonmedical treatments of vocal fold nodules: a systematic review. Journal of Voice.

[33] Lukaschyk J, Brockmann-Bauser M, Beushausen U (2017). Transcultural Adaptation and Validation of the German Version of the Vocal Tract Discomfort Scale. Journal of Voice.

